# An Elevated Glycemic Gap is Associated With Adverse Outcomes in Diabetic Patients With Community-Acquired Pneumonia

**DOI:** 10.1097/MD.0000000000001456

**Published:** 2015-08-28

**Authors:** Po-Chuan Chen, Wen-I. Liao, Ying-Chuan Wang, Wei-Chou Chang, Chin-Wang Hsu, Ying-Hsin Chen, Shih-Hung Tsai

**Affiliations:** From the Department of Emergency Medicine (PCC, WIL, YHC, SHT); Department of Family Medicine (YCW); Department of Radiology, Tri-Service General Hospital, National Defense Medical Center (WCC); and Department of Emergency and Critical Care Medicine, Taipei Medical University-Wan Fang Hospital, Taipei, Taiwan (CWH).

## Abstract

Several studies argue against the association between admission hyperglycemia and adverse outcomes in infected diabetic patients. When investigating the association, it is necessary to consider preexisting hyperglycemia. The objective of this study was to assess whether stress-induced hyperglycemia, determined by the glycemic gap between admission glucose levels and A1c-derived average glucose levels adversely affects outcomes in diabetic patients admitted to hospital with community-acquired pneumonia (CAP).

We retrospectively analyzed the glycemic gap and adverse outcomes of diabetic patients hospitalized because of CAP from June 1, 2007 to August 31, 2012 in single medical center in Taiwan.

A total of 203 patients admitted with principal diagnosis of CAP and available data of glycemic gap.

Patients with glycemic gaps ≥40 mg/dL had greater AUROC values for the development of adverse outcomes compared with acute hyperglycemia and long-term glycemic controls. Patients with an elevated glycemic gap had an odds ratio of 3.84 for the incidence of combined adverse outcomes. Incorporation of the glycemic gap into pneumonia severity index, CURB-65 or SMART-COP scores, increased the discriminative performance of predicting the development of adverse outcomes.

Glycemic gaps were associated with adverse outcomes of diabetic CAP patients. The discriminative performance of the calculated glycemic gaps was comparable with those of current clinical scoring systems and may further increase the AUROC of each system.

## INTRODUCTION

Community-acquired pneumonia (CAP) is the leading infectious cause of death in developed countries. Many predisposing factors can influence the prognosis of patients with CAP. Diabetes mellitus (DM) may inhibit the defense functions of the host's histiocytic cells, such as chemotaxis, phagocytosis, and bactericidal activity.^1^ Stratifying the severity and prognosis of CAP is very important for making treatment decisions in daily emergency practice. Severity assessment scores are used to determine whether patients require hospitalization or admittance to the intensive care unit (ICU). The pneumonia severity index (PSI) and CURB-65 (confusion, urea > 7 mmol/L, respiratory rate > 30/min, low systolic [<90 mmHg] or diastolic [<60 mmHg] blood pressure, and age ≥65 years) were developed to assess the severity of pneumonia and predict 30-day mortality rates with good sensitivity and specificity.^2–5^ Further, the SMART-COP tool that takes into account low systolic blood pressure, multilobar chest radiographic involvement, low albumin level, high respiratory rate, tachycardia, confusion, poor oxygenation, and low arterial pH was developed to predict the requirements for intensive respiratory and vasopressor support as well as admission to the ICU.^6^

Stress-induced hyperglycemia (SIH) commonly occurs in patients with critical illnesses such as sepsis, multiple trauma, burn injuries, and myocardial infarction.^7^ For example, 1 study found that 67% of patients hospitalized with pneumonia had SIH.^8^ Moreover, acute and mean hyperglycemia during hospitalization are associated with adverse clinical outcomes.^9–11^ However, there are discordant findings about the correlation between hyperglycemia and adverse outcomes in acutely ill patients with or without preexisting diabetes.^12–14^ Several studies argue against the association between hyperglycemia upon admission and adverse outcomes in infected diabetic patients.^15^

A strong correlation exists between glycated hemoglobin A1c (HbA1c) and long-term mean plasma glucose levels in the preceding 3 months. The results of an international multicenter study of HbA1c-derived average glucose allows estimation of long-term average glucose levels using HbA1c values.^16^ Because hyperglycemia is the cardinal feature of diabetes, it is necessary to consider preexisting hyperglycemia in diabetic patients when investigating the association between SIH and adverse outcomes. We therefore speculated that the fundamental question is what causes acute serum glucose levels. In acutely ill diabetic patients, the epiphenomenon of admission hyperglycemia may be caused by acute physiological stress, higher chronic baseline blood glucose levels, or both.^15^

The aim of the present study was to further explore the correlations among glycemic gaps, 3 validated clinical scoring systems, and adverse clinical outcomes in patients with both diabetes and CAP and to justify the use of the glycemic gap as a biomarker for the assessment of the severity of pneumonia.^17^

## MATERIALS AND METHODS

### Patients

The institutional review board for human investigations of a tertiary referral medical center in northern Taiwan approved this study and waived informed consent. We conducted a retrospective observational study of all patients with DM admitted for CAP between June 1, 2007 and August 31, 2012. The identification of patients with DM and pneumonia was performed by searching the International Classification of Diseases (9th revision) codes 486.0 and 250.2–8. The patients were then reviewed to select those patients with CAP with data for plasma glucose levels at initial presentation and HbA1c levels within 1 month before or after to their admission. Patients with concurrent infections, use of steroids, or hypoglycemia (blood glucose < 70 mg/dL) were excluded. CAP was diagnosed if at least 1 symptom of acute lower respiratory infection was accompanied by new radiographic evidence. Patients were excluded if they developed pneumonia 48 hours after admission or within 2 weeks after discharge from a hospital. Antibiotic treatment of CAP generally complied with the consensus guidelines of the Infectious Diseases Society of America/American Thoracic Society for the management of CAP in adults.^18–20^ A diagnosis of diabetes was confirmed if a patient was discharged from a hospital with a diagnosis of either type 1 or type 2 diabetes, and/or treated with insulin or an oral antidiabetic agent, and/or had an HbA1c level ≥6.5% 2 months prior to the index date.^21,22^ The diagnosis and treatment of acute respiratory distress syndrome (ARDS) and using a ventilator generally complied with the American–European Consensus Conference definition as the acute onset of impaired gas exchange and the presence of bilateral alveolar or interstitial infiltrates in the absence of congestive heart failure as well as the recommendations of the Surviving Sepsis Campaign.^23–26^

## METHODS

We retrospectively reviewed patients’ medical records to determine age, sex, underlying comorbidities, clinical presentation, laboratory data, including plasma glucose level at initial presentation, HbA1c levels (measured within 1 month before or after admission), adverse outcomes, length of mechanical ventilation, and stays in the ICU and the hospital. Adverse outcomes were as follows: mortality during admission; acute respiratory distress syndrome (ARDS, acute decrease in the ratio of partial pressure of arterial oxygen to fraction of inspired oxygen to 300 or less, bilateral pulmonary infiltrates on a chest radiograph consistent with the presence of edema, and no clinical evidence of left atrial hypertension);^27^ acute respiratory failure (ARF) that required ventilation support; failure of weaning from a ventilator (defined as administering mechanical ventilation during discharge); septic shock (defined as sepsis-induced persistent hypotension despite adequate fluid resuscitation);^28^ pulmonary complications (defined as development of empyema, pulmonary abscess, or pleura effusion); bacteremia; acute kidney injury (AKI, defined as serum creatinine elevated > 0.3 mg/dL or 50% from baseline);^29^ upper gastrointestinal bleeding (UGIB, defined as melena with positive occult blood examination, bright-red blood discharged from the nasogastric tube, or endoscopic evidence of mucosal bleeding); and acute myocardial infarction (AMI, clinical and laboratory evidence of myocardial necrosis and clinical findings of ischemia were present in accordance with global taskforce recommendation.)^30^ during hospitalization. We recorded the clinical data that were required to calculate PSI, CURB-65, and SMART-COP to further quantify the severity of pneumonia.

### Measurements of Serum Glucose and HbA1C

The glucose level upon admission was defined as that determined upon initial presentation to the Emergency Department. HbA1c assays were performed at Tri-Service General Hospital using a blood analyzer (Primus CLC 385; Primus Corporation, Kansas City, MO) equipped with a high-performance liquid chromatography system. The laboratory received level-1 certification for conducting this analysis from the National Glycohemoglobin Standardization Program.

The equation AG = 28.7 × HbA1c-46.7 was used to convert HbA1c levels to estimated long-term average glucose levels over the past 3 months.^16^ The glycemic gap, which represents changes of serum glucose levels during this event, was calculated from the glucose level upon admission minus estimated long-term average glucose levels.

### Statistical Analysis

Categorical data are presented as frequencies (%) and were evaluated using the Chi-square/Fisher exact test. Continuous data are expressed as the mean ± standard deviation and analyzed using the 2-tailed Student *t*-test. One-way analysis of variance was used to assess the significance of various clinical characteristics, laboratory data, and adverse outcomes. Post-hoc analysis was performed using the Bonferroni test. A receiver–operator characteristic curve (ROC) was created to analyze the discriminative power of the prediction rules; and the area under receiver–operator characteristic curve (AUROC) and 95% confidence intervals (CI) were subsequently calculated. The data were analyzed using the Statistical Package for the Social Sciences version 17.0 statistical software (SPSS, Inc., Chicago, IL). Differences were considered statistically significant when the *P* values < 0.05. The Youden index was used to determine the optimal cut-off value for discriminative power of the ROC. The net reclassification improvement (NRI) was used to assess the improvement in model performance after adding parameters (MATLAB, MathWorks, Natick, MA).^31^

## RESULTS

### Patient Study Population and Clinical Outcomes

We initially identified 746 patients with the admission diagnosis of CAP along and type 2 diabetes. Patients were excluded because, hypoglycemia upon admission (n = 121), and incomplete data, use of steroid and anemia and received blood transfusion (n = 422). We enrolled 203 patients after subsequent chart review. Demographic data and CAP-related clinical features (including PSI, CURB-65, and SMART-COP scores) of the enrolled patients are shown in Tables 1 and 2. Of these patients, 20 (9.9%) died during hospitalization, 62 (30.5%) were admitted to the ICU, 42 (20.7%) required mechanical ventilation, 16 (7.9%) were weaning failures, 8 (3.9%) developed ARDS, 29 (14.3%) developed septic shock, 15 (7.4%) had bacteremia, 72 (35.5%) experienced AKI, 40 (19.7%) developed pulmonary complications, 11 (5.4%) developed AMI, and 33 (16.3%) developed UGIB.

**TABLE 1:**
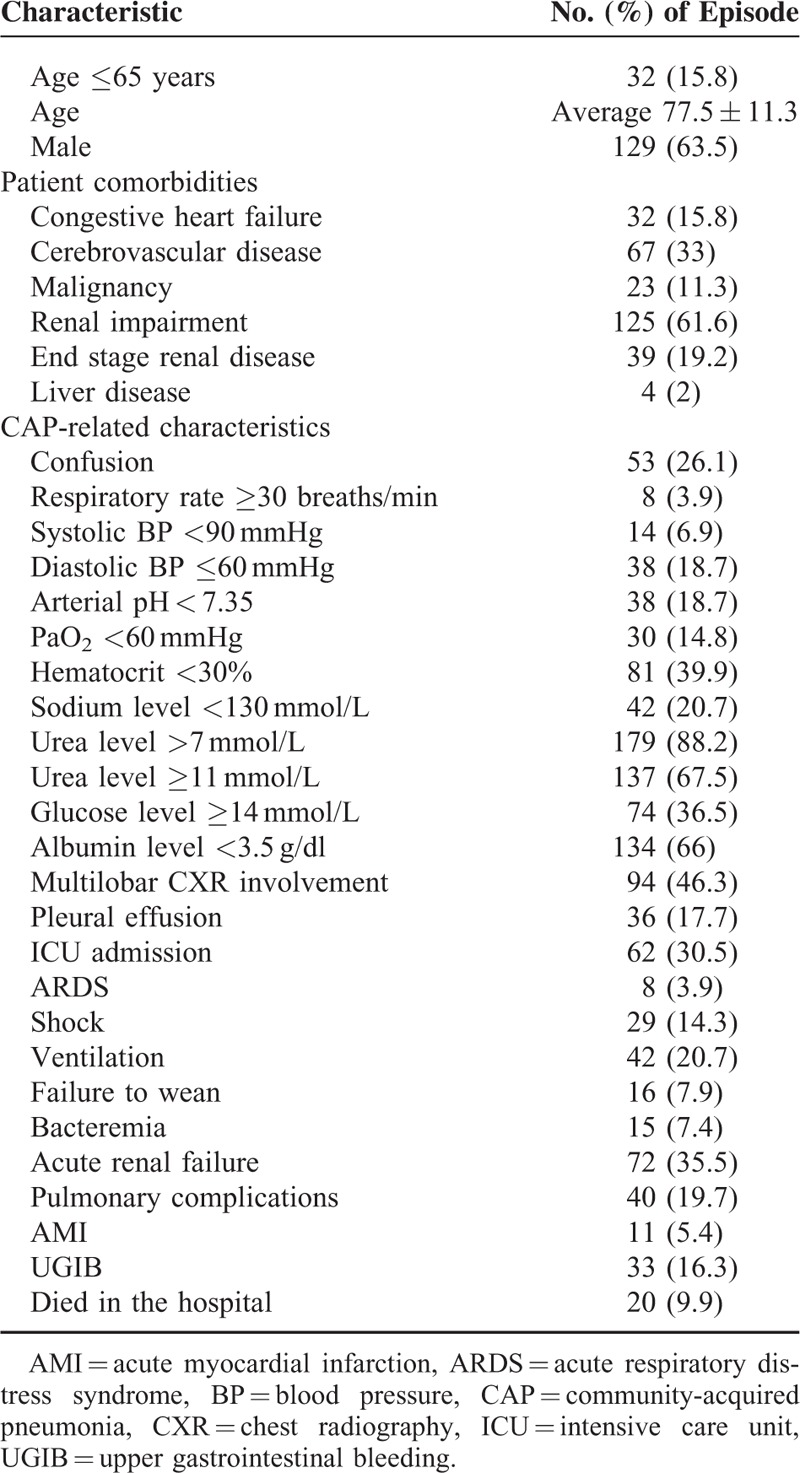
Baseline Characteristics of Diabetic Patients Who Experienced Episodes of Community-Acquired Pneumonia

**TABLE 2:**
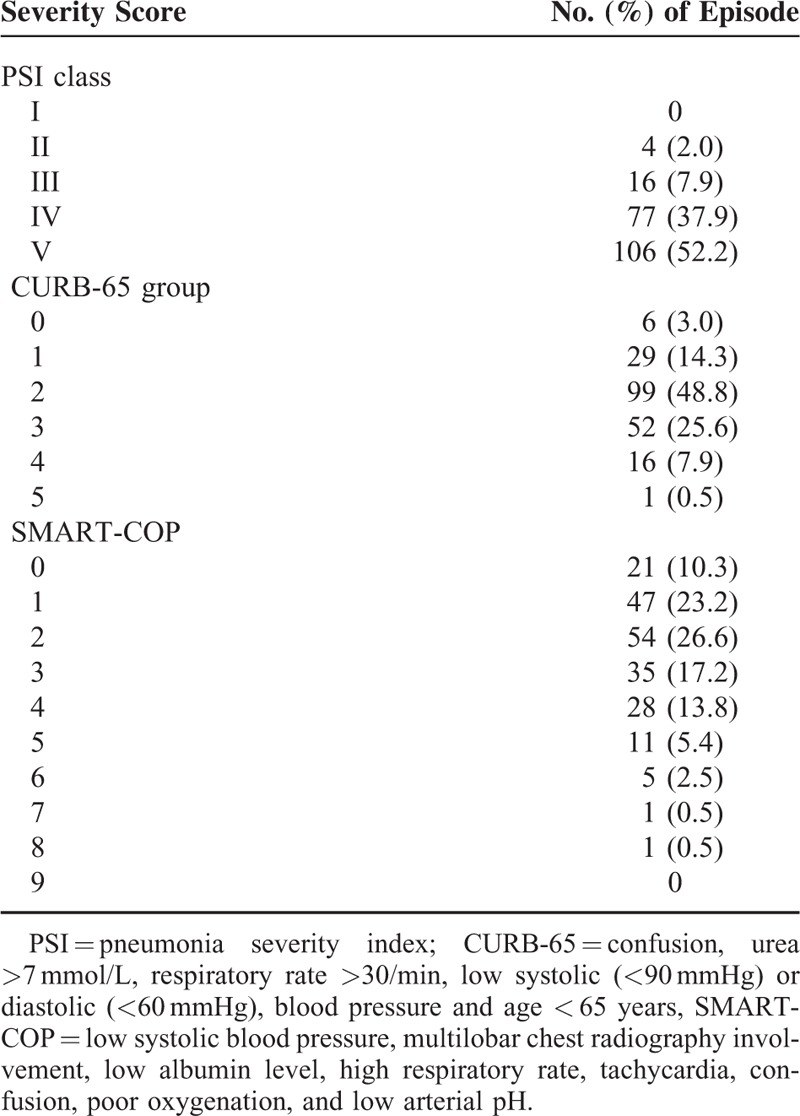
Baseline Severity of Diabetic Patients Who Experienced Episodes of Community-Acquired Pneumonia

### Correlations Among Acute Hyperglycemia, Glycemic Gaps, Long-Term Blood Glucose Control, and Adverse Outcomes

**FIGURE 1:**
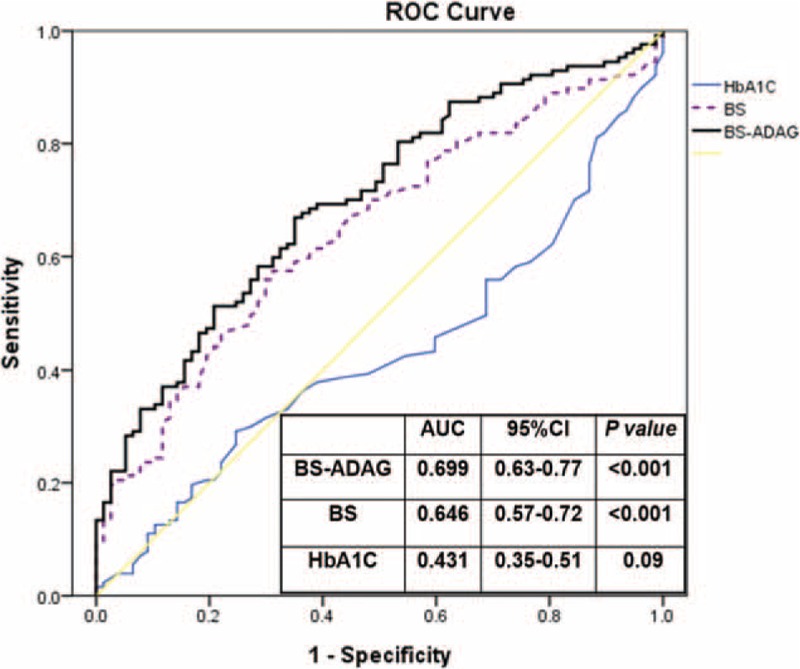
ROC of acute hyperglycemia, chronic blood glucose controls, glycemic gaps, and adverse outcomes. AUC = area under the curve, ROC = receiver operating characteristic.

Patients with acute hyperglycemia, which is defined as blood glucose level of ≥250 mg/dL, the value used to calculate PSI, were associated with increased combined adverse outcomes, the development of AKI, septic shock, and ICU admission with longer ICU and total hospital stays (Table 3). However, compared with acute hyperglycemia (AUROC = 0.646, [95% CI = 0.57–0.72]) and long-term glycemic controls (AUROC = 0.431, [95% CI = 0.35–0.51]), glycemic gaps (AUROC = 0.699, [95% CI = 0.63–0.77]) had greater AUROC values for the development of adverse outcomes (Figure 1). We then determined an optimal cutoff value of 40 mg/dL by using the Youden index, with sensitivity and specificity of 66.9% and 64.9%, respectively, for the development of adverse outcomes. There was no statistically significant difference between comorbidity among patients with or without an elevated glycemic gap. Compared with patients with diabetes and CAP who had a glycemic gap of <40 mg/dL (44.8%), those with an elevated glycemic gap of ≥40 mg/dL (55.2%) had an odds ratio of 3.84 for the incidence of combined adverse outcomes (*P* < 0.01) (Table 4). Further analysis revealed that patients with an elevated glycemic gap had an increased risk of AKI (*P* < 0.01), ARF (*P* < 0.01), septic shock (*P* < 0.05), and admission to the ICU (*P* < 0.01). They had a statistically significant greater number of days of mechanical ventilation (*P* < 0.05) and length of ICU and hospital stays (*P* < 0.001 and 0.05, respectively). We found that chronic glycemic controls affected the adverse outcomes, bacteremia, and length of ICU and hospital stays (Table 5). Patients with good glycemic control had a higher risk for adverse outcomes and longer ICU and hospital stays (*P* = 0.01, 0.037, and 0.026, respectively). Poorer glycemic control (HbA1c values of >9%) was not associated with adverse outcomes.

**TABLE 3:**
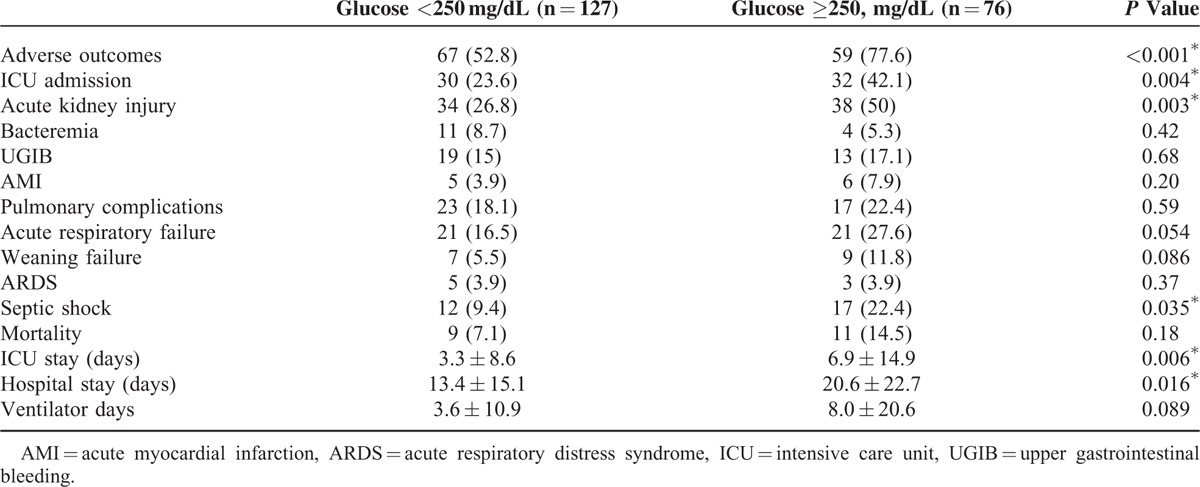
Clinical Outcomes Versus Acute Hyperglycemia in Patients with Both Diabetes and Community-Acquired Pneumonia

**TABLE 4:**
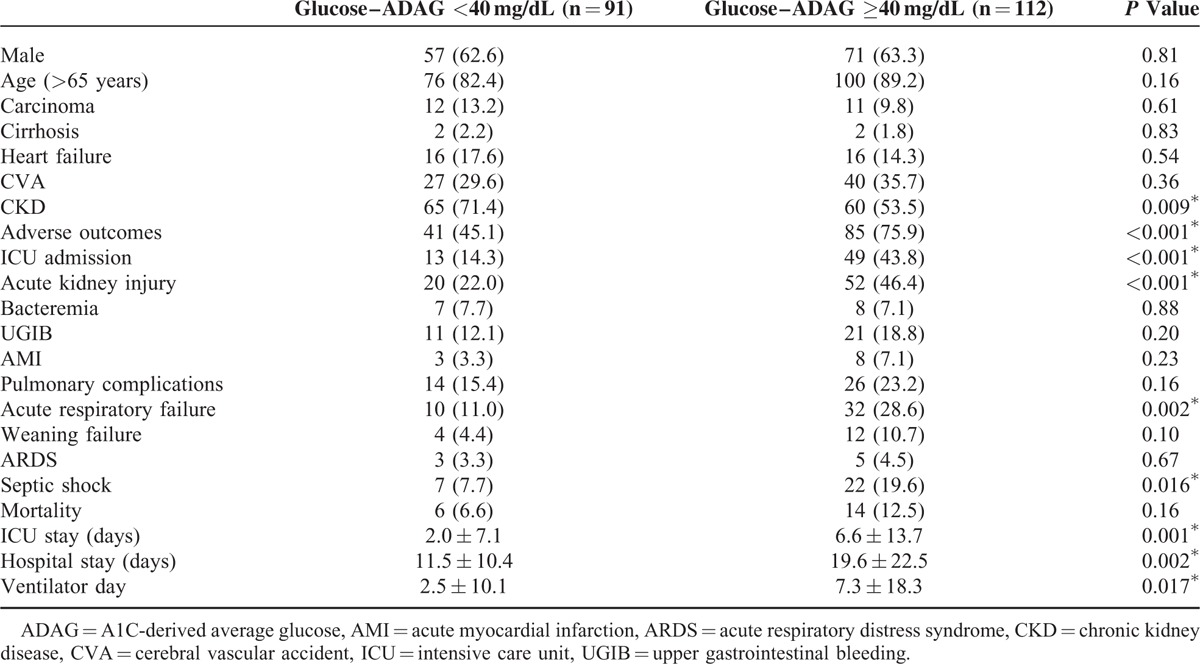
Characteristic and Clinical Outcome Versus Glycemic Gap of Patients With Both Diabetes and Community-Acquired Pneumonia

**TABLE 5:**
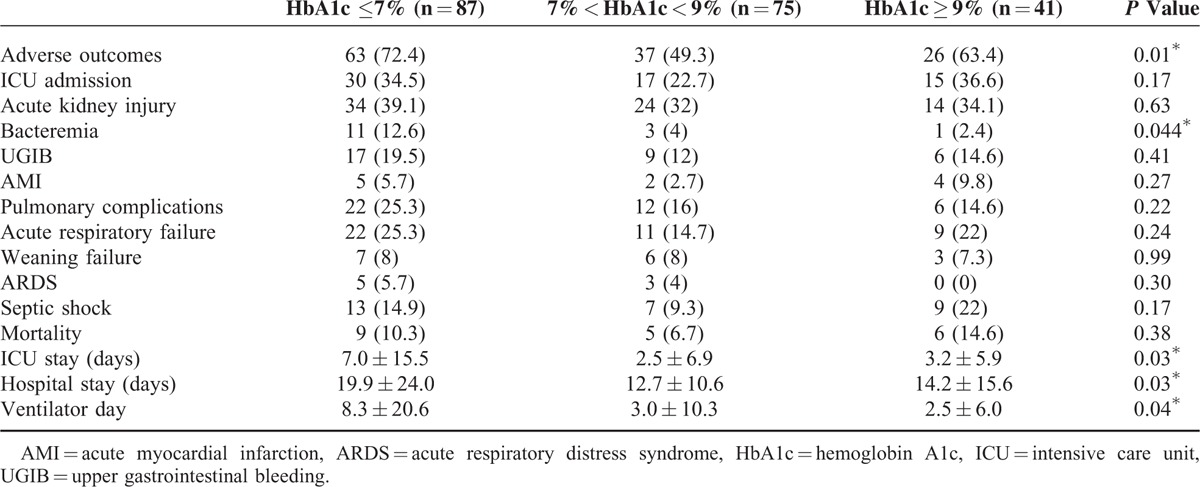
Clinical Outcomes Versus Chronic Glycemic Control in Patients With Both Diabetes and Community-Acquired Pneumonia

### Association Between the Glycemic Gap and CAP Clinical Scores

**FIGURE 2:**
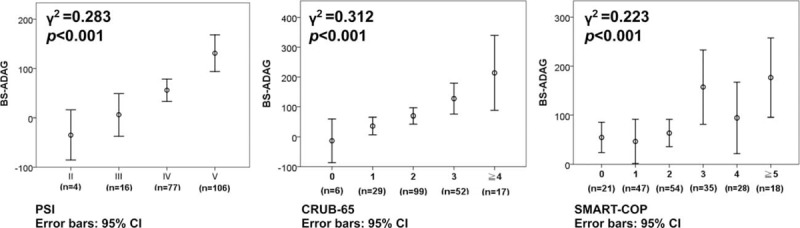
Correlations between the glycemic gaps and community-acquired pneumonia-associated clinical scoring systems. CURB-65 = confusion, urea >7 mmol/L, respiratory rate > 30/min, low systolic (<90 mmHg) or diastolic (<60 mmHg) blood pressure, and age ≥65 years, SMART-COP = low systolic blood pressure, multilobar chest radiographic involvement, low albumin level, high respiratory rate, tachycardia, confusion, poor oxygenation, and low arterial pH, PSI = pneumonia severity index.

**FIGURE 3:**
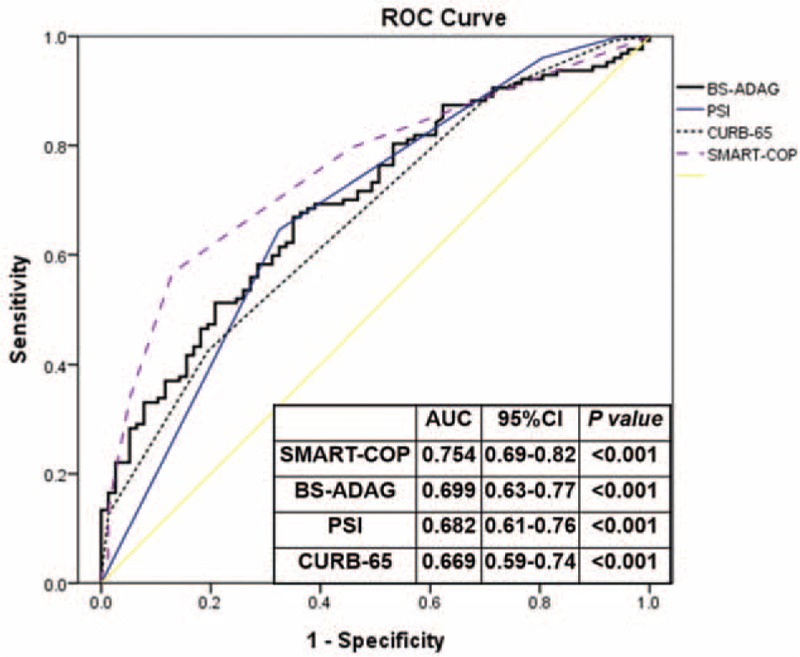
ROC of the glycemic gap, pneumonia severity index, CURB-65, and SMART-COP and community-acquired pneumonia associated adverse outcomes. ADAG = A1C-derived average glucose, AUC = area under the curve, CURB-65 = confusion, urea >7 mmol/L, respiratory rate > 30/min, low systolic (<90 mmHg) or diastolic (<60 mmHg) blood pressure, and age ≥65 years, , PSI = pneumonia severity index, ROC = receiver operating characteristic, SMART-COP = low systolic blood pressure, multilobar chest radiographic involvement, low albumin level, high respiratory rate, tachycardia, confusion, poor oxygenation, and low arterial pH.

There was a statistically significant correlation between the levels of glycemic gaps and PSI, CURB-65, and SMART-COP scores but with a low correlation coefficient (Figure 2). The AUROCs for each severity assessment tool are shown in Figure 3. Although the SMART-COP score had the greatest AUROC (0.754, [95% CI = 0.69–0.82]), the discriminative power of glycemic gaps was greater for adverse outcomes compared with those of PSI and CURB-65 (AUROC = 0.699 [95% CI = 0.63–0.77] vs 0.682 [95% CI = 0.61–0.76] vs 0.669 [95% CI = 0.59–0.74], respectively).

**FIGURE 4:**
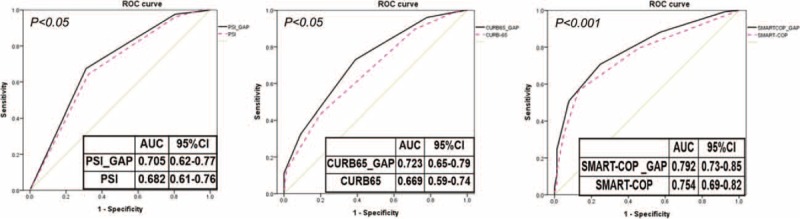
The effects of integrating glycemic gaps into the PSI, CURB-65, and SMART-COP. AUC = area under the curve, CURB-65 = confusion, urea > 7 mmol/L, respiratory rate > 30/min, low systolic (<90 mmHg) or diastolic (<60 mmHg) blood pressure, and age ≥65 years, PSI = pneumonia severity index, ROC = receiver operating characteristic, SMART-COP = low systolic blood pressure, multilobar chest radiographic involvement, low albumin level, high respiratory rate, tachycardia, confusion, poor oxygenation, and low arterial pH.

Incorporation of the glycemic gap, either by replacing the hyperglycemia value of >250 mg/dL with that of the glycemic gap of >40 mg/dL in PSI score or by adding the glycemic gaps into the CURB-65 or SMART-COP scores, statistically significant increased the discriminative performance of predicting the development of adverse outcomes by increasing the AUROC from 0.682[95% CI = 0.61–0.76] to 0.705[95% CI = 0.62–0.77, NRI = 0.031, *P* = 0.045], 0.669[95% CI = 0.59–0.74] to 0.723[95% CI = 0.65–0.79, NRI = 0.238, *P* = 0.0012], and 0.754 [95% CI = 0.69–0.82] to 0.792 [95% CI = 0.73–0.85, NRI = 0.167, *P* = 0.0001], respectively (Figure 4).

## DISCUSSION

The major findings of the present study are as follows: elevated glycemic gaps associated with the development of adverse outcomes such as ARF, AKI, septic shock, ICU admission, longer hospital and ICU stays, and ventilator days; the glycemic gap had better discriminative performance than acute hyperglycemia for the development, rather than long-term blood glucose controls, predicted CAP-related adverse outcomes of diabetic patients; and adding the glycemic gaps into the PSI, CURB-65, and SMART-COP further increased their discriminative performance for predicting adverse outcomes. These findings suggest that glycemic gap could be incorporated into future clinical scoring systems to enhance their discriminative performance.

SIH is attributed to the presence of excess levels of counter-regulatory hormones, antiinflammatory cytokines, and increased gluconeogenesis and hepatic insulin resistance.^7,32,33^ DM is a strong predictor of the risk of mortality from pneumonia for seniors (age ≥65-years old).^34^ Whether DM and acute hyperglycemia influence the outcomes of certain infectious diseases, including pneumonia, is controversial.^34–38^ Several studies indicate that acute hyperglycemia correlates with poorer outcomes in nondiabetic patients.^39^ Acute hyperglycemia is associated with increased severity and adverse outcomes of patients with trauma,^40–42^ unfavorable neurological improvement and symptomatic hemorrhage in thrombolytic therapy treated acute ischemic stroke patients,^43^ increased severity and risk of nosocomial complications in patients with CAP,^15,44,45^ and major adverse cardiac events in patients with AMI.^46^

We previously published a proof-of-concept article about using glycemic gaps to eliminate the influence of chronic hyperglycemia on the evaluation of admission hyperglycemia in diabetic patient with liver abscess. We had found that an elevated glycemic gap of >72 mg/dL, rather than admission hyperglycemia or chronic glycemic control, significantly correlated with adverse outcomes.^47^ In this study, we again found that the glycemic gap at 40 mg/dL had comparable discriminative performance for distinguishing among the development of CAP-related adverse outcomes in diabetic patients. We speculated that the lower cut-off value for the glycemic gaps in this CAP study was due to the nature of the disease and spectrum of the severity was much wider in CAP than in liver abscess, that is, smaller glycemic gaps were needed to discriminate the difference.

HbA1c levels are a better index of overall glycemic exposure, is characterized by lower biological variability, and is relatively unaffected by acute stress or sepsis, since it would not have time to “catch-up” with acute elevation.^22^ The consistent findings regarding the association between elevated glycemic gaps and adverse outcomes in diabetic patients with liver abscess and CAP allowed us to strengthen our hypothesis that an acute surge of glucose levels beyond the long-term average (or in nondiabetic patients) serves as a surrogate marker for acute physiological stress. The glycemic gap reflects “additional” glucose homeostasis in response to physical stress on chronic glycemic control. We believe that the glycemic gap explains the “diabetes paradox” and debates about the association between acute hyperglycemia, long-term glucose controls, and certain adverse clinical outcomes.^48^

There is considerable clinical and research interest in the use of biomarkers or clinical scoring systems to diagnose and classify CAP. Initial triage of patients with severe CAP may result in better outcomes.^49^ Various clinical severity scoring systems and biomarkers were developed to predict adverse events, ICU admission, treatment failure, and mortality. Patients with higher CRP and procalcitonin levels are at higher risk for bacteremia, complications, and longer hospitalization.^50–52^ Most studies report that incorporation of another biomarker of inflammation into the clinical scoring system increases the AUROC for adverse outcomes.^53^ In contrast, other studies argue against the association between biomarkers of inflammation and outcomes.^54,55^ Moreover, cost-effectiveness is another concern for using such biomarkers.^56,57^ It was suggested that when a novel biomarker becomes available to help facilitate risk prediction, it is essential to measure the improvement compared with the existing practice tool, that is, combining different biomarkers and clinical scores to further increase the AUROC.^3,58^ In NRI analysis, we found that incorporation the glycemic gaps into the PSI, CURB-65, and SMART-COP scores could significantly increase the AUROC of each prediction rule. The American Diabetes Association currently recommends biannual evaluation of HbA1c levels of patients who are meeting treatment targets and have stable glycemic controls, or quarterly evaluation in patients whose therapy has changed or who are not meeting glycemic targets.^21^ Thus, we believe that the integration of HbA1c data in the assessment of acute infectious diseases is clinically feasible and may provide a severity index without adding more laboratory tests.

Studies on how chronic glucose control affects the outcomes of acute infectious episodes are still controversial. Poorer chronic glycemic control was associated with increased risk of pneumonia-related hospitalization.^59^ In addition, poorly controlled diabetes was also correlated with a greater incidence of adverse outcomes and significantly longer hospital stays for surgical patients;^60,61^ increased risk of hospitalization for heart failure,^62^ unfavorable neurological and functional outcomes of in patients with acute ischemic stroke,^63^ higher risk of amputation for patients with critical limb ischemia,^64^ and ventilator-associated pneumonia and septicemia in critically ill patients.^65^ Nonetheless, diabetes is associated with a lower rate of developing ARDS, and this relationship remains after adjusting for clinical differences between diabetics and nondiabetics.^36^ Egi et al^14^ demonstrated that preexisting glycemic control may alter the association between acute hyperglycemia and mortality in critically ill diabetic patients. Chronic hyperglycemia assessed by HbA1c did not predict infarct size and in-hospital mortality in patients with AMI.^66^ In the present study, we also found that patients with poorer glycemic control were not at increased risk of CAP-related adverse outcomes.

## LIMITATIONS

Our study has several limitations. First, it was retrospective and may have been subject to selection bias. Second, the adequacy of glycemic control during hospitalization may have influenced the outcomes.^39^ The trigger to start an insulin protocol is currently a blood glucose level of 180 mg/dL.^25,67^ In present study, we did not specifically address the effects of glycemic controls during hospitalization. Nonetheless, recent studies suggest that attempts at tight glycemic control do not improve outcomes.^68^ Future studies need to control for this factor in a subgroup analysis in light of the findings.

## CONCLUSION

Glycemic gaps between admission serum glucose levels and HbA1c-derived average glucose were associated with adverse CAP-related outcomes. The discriminative performance of the glycemic gaps was comparable with those of current clinical scoring systems and may further increase the AUROC of each system. We conclude that the glycemic gaps can be used to assess the severity and prognosis of certain acute illness in diabetic patients.
